# The effect of a single-strain probiotic administration in the treatment of thermal burns patients

**Published:** 2019-06

**Authors:** David S. Perdanakusuma, Lynda Hariani, Nur Febriany Nasser, Robertus Arian Datusanantyo

**Affiliations:** Department of Plastic Reconstructive and Aesthetic Surgery, School of Medicine, Universitas Airlangga, Dr. Soetomo General Hospital, Surabaya, Indonesia

**Keywords:** Probiotic, Microbiome, Bacterial translocation, *Lactobacillus*, *Bifidobacterium*

## Abstract

**Background and Objectives:**

Between 2007 and 2011, the mortality rate for burns patients at Dr. Soetomo General Hospital, Surabaya, Indonesia was 14.1% and 60% were suspected to be sepsis-related. Immunosuppression, gut barrier disruption, and intestinal hypomotility cause bacterial and bacterial product translocation. Probiotics improve the intestinal microbiome and eventually reduce bacterial translocation, and an increased secretory immunoglobulin A (SIgA) secretion post-administration of a multi-species probiotic has been observed. We aimed to determine whether a single-strain probiotic administration could show strengthened intestinal immunity, through an increase in SIgA levels, as with multi-strain probiotics.

**Materials and Methods:**

Sixteen burns patients from our hospital Burns Centre were randomized into three treatment groups, and the patients were administered either a placebo, a *Lactobacillus reuteri protectis* probiotic, or a *Bifidobacterium infantis* 35624 probiotic for 14 consecutive days. The SIgA levels were analyzed using ELISA pre- and post-treatment.

**Results:**

The post-treatment SIgA levels in the placebo, *Lactobacillus reuteri protectis* probiotic, and *Bifidobacterium infantis* 35624 probiotic groups were 222.56±74.22 mg/dL, 223.92±68.89 mg/dL, and 332.38±64.27 mg/dL, respectively. Decreased SIgA levels were observed in the placebo (7.19±15.87) and in the *Lactobacillus reuteri protectis* probiotic (1.9920±14.76) groups, whereas an increase was seen in the SIgA level in the *Bifidobacterium infantis* 35624 probiotic group (58.26±77.41).

**Conclusion:**

The *Bifidobacterium infantis* 35624 single-strain probiotic is generally superior to *Lactobacillus reuteri protectis* in altering intestinal immunity; however, this finding was not statistically significant. A multi-strain probiotic supplement is recommended for burns patients.

## INTRODUCTION

The end result of hemodynamic changes due to burns is a decreased blood flow (1). In rat burn wound models, bacterial overgrowth of *Enterobacteriaceae* (*Escherichia, Klebsiella, Proteus* and *Citrobacter*) has been observed, as with many sepsis burns patients. Post-burn gene expression alteration was found to be related to microbiome change, gut barrier integrity disruption, and pathogenic opportunistic bacterial invasion (2), leading to translocation of bacteria and bacterial products from the gut to the blood circulation resulting in sepsis, multi-organ dysfunction syndrome, and death (3).

At Dr. Soetomo General Hospital in Surabaya, Indonesia between 2007 and 2011, the mortality rate for burns patients was 14.1% and 60% of burns-related deaths were suspected to be sepsis-related (4).

Probiotics are living organisms that, when administered in adequate amounts, confer a health benefit for the host beyond a basic nutritional role (5) and are administered to overcome microbiome change and bacterial translocation in burns patients. Probiotic health benefits have previously been reported, such as increasing mucosal immunity (6), despite conflicting evidence concerning their effects on burns patients’ hospital length of stay (7, 8). However, some studies have shown improved wound healing rates for burns patients post-administration of a probiotic (7–9). Despite a decreasing tendency for infection (8), probiotic administration has not been shown to prevent sepsis (9).

A previous study undertaken at our hospital examined 33 burns patients and found that fecal secretory immunoglobulin A (SIgA) levels increased 61.25% post-administration of a multi-strain probiotic, namely Protexin®, comprised of *Lactobacillus casei, Lactobacillus rhamnosus, Lactobacillus acidophilus, Lactobacillus bulgaricus, Bifidobacterium breve, Bifidobacterium longum* and *Streptococcus thermophilus*, whereas a decreased fecal SIgA level of 35.8% was found in a group that received no probiotic (Wahyudi & Noer, 2010 – unpublished data). That study prompted the question as to whether a single-strain probiotic would increase the SIgA level in a manner similar to a multi-strain probiotic.

Herein, We aimed to determine whether a single-strain probiotic administration could demonstrate strengthened intestinal immunity, through an increase in SIgA levels, as with multi-strain probiotics.

## MATERIALS AND METHODS

This study was a randomized double-blind controlled trial between April and September 2013. Inclusion criteria involved patients aged between 16 and 60 years old who had sustained a burn injury of ≥10% total body surface area, who presented at our hospital within 24 hours of the burn injury. Exclusion criteria involved patients who had taken a probiotic prior to admission and sepsis.

All patients who met the inclusion criteria were randomized into three treatment groups. The first group received a placebo, the second group received 1 × 10^9^ (1 billion) (4 mg) live bacteria/colony forming units (CFU) *Bifidobacterium infantis* 35624 (Bifantis Align®), and the third group received 1 × 10^8^ CFU *Lactobacillus reuteri protectis* (Rillus®). The treatment was administered on the fourth day post-burn injury, once daily, for 14 consecutive days.

Fecal samples for SIgA measurements were obtained pre- and post-administration of the probiotics. SIgA levels were measured using an enzyme-linked immunosorbent assay (ELISA) method at the Immunology-Serology Laboratory, Institute of Tropical Disease, Universitas Airlangga, Surabaya.

Each study patient provided their written consent after having received information concerning the study from the researchers. Ethical approval was obtained from the Ethical Committee of Dr. Soetomo General Hospital Surabaya (No. 223/Panke.KKE/VIII/2013).

## RESULTS

A total of 16 patients met the inclusion criteria and these patients were randomized into three treatment groups. There were more males (n=12, 75%) than females (n=4,25%); however, this was not statistically significant (p=0.589). The mean age of the patients in the placebo, *Bifidobacterium* and *Lactobacillus* groups was 33.2 years (range, 16–50 years), 40. 2 (range, 24–58 years), and 25.2 years (range, 16–37 years), respectively. There was no statistical difference in the mean age between the groups (p=0.605).

The burn wound percentages in the placebo, *Bifidobacterium* and *Lactobacillus* groups were 26.1% (n=5; range, 5%–58%); 21.83% (n=6; range, 5%–32%); and 31.4% (n=5; range, 5%–80.5%), respectively. There was no statistical difference in the percentage of burn wounds (p=0.721) between the groups. Most patient admissions were due to electric burn injuries (40%).

Pre-treatment, fecal SIgA levels in the placebo, *Lactobacillus*, and *Bifidobacterium* groups were 229.76±61.08 mg/dL, 225.91±81.63 mg/dL and 274.13±8395 mg/dL, respectively, although the SIgA level differences were not statistically significant (p=0.524).

Post-treatment, fecal SIgA levels in the placebo, *Lactobacillus* and *Bifidobacterium* groups were 222.56±74.22 mg/dL, 223.92±68.89 mg/dL, and 332.38±64.27 mg/dL, respectively. An ANOVA statistical analysis showed a significant difference among the groups (p=0.029). Significant differences were observed between the placebo and *Bifidobacterium* groups (p=0.027) and between the *Lactobacillus* and the *Bifidobacterium* groups (p=0.024).

**Fig. 1.**
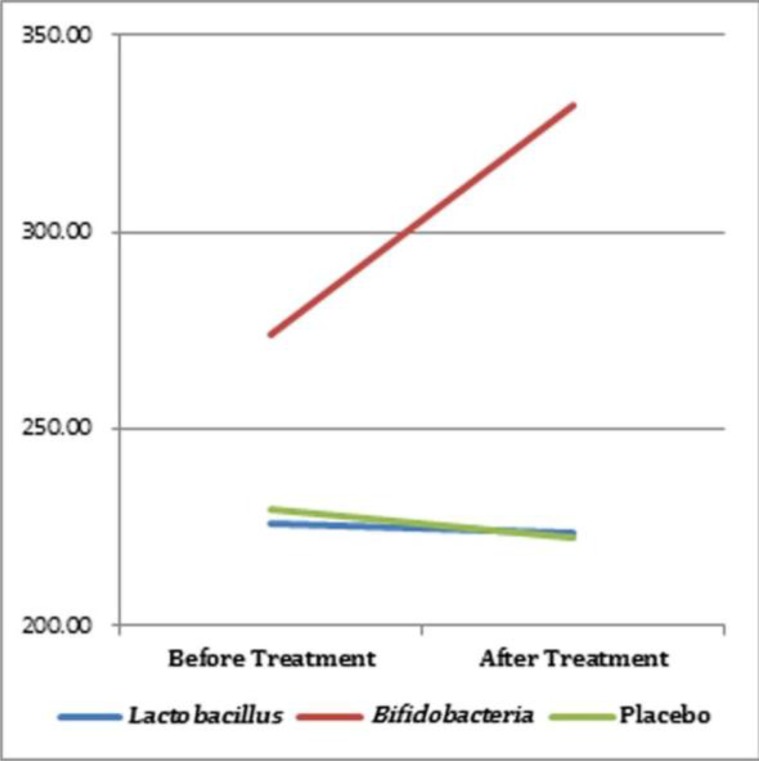
SIgA levels pre- and post-treatment

The decrease in SIgA levels was 7.19±15.87 mg/dL in the placebo group and 1.9920±14.76 mg/dL in the *Lactobacillus* group, whereas there was an increase in the SIgA levels in the *Bifidobacterium* group (58.26±77.41 mg/dL); however, these differences were not statistically significant (p=0.083). The SIgA levels are summarized in Table 1 and shown in Fig. 1.

**Table 1.** SIgA levels for all three treatment groups

	**Placebo (mg/dL)**	***Lactobacillus* (mg/dL)**	***Bifidobacterium* (mg/dL)**	**p-value**
Pre-treatment	229.76±61.08	225.91±81.63	274.13±83.95	0.524
Post-treatment	222.56±74.22	223.92±68.89	332.38±64.27	0.029
Difference	−7.19±15.87	−1.9920±14.76	58.26±77.41	0.083

## DISCUSSION

Systemic inflammation due to burns not only results in increased endothelial cell permeability but also in increased permeability in other epithelial cells such as the intestinal epithelial cells (10). This condition is related to bacterial translocation in burns. Moreover, an increased possibility of bacterial translocation is also due to a decrease in the adaptive immune response and in intestinal hypomotility (11, 12).

Probiotic benefits in our study, and in a previous study (Wahyudi & Noer, 2010 – unpublished data) undertaken at our hospital, were assessed through determining the increased SIgA levels post-administration of probiotics in burns patients. We opted to determine the SIgA level as SIgA may inhibit bacterial attachment and invasion to epithelial cells of the gut mucosa, with the end result being bacterial translocation prevention in burns patients (13).

The previous study (Wahyudi & Noer, 2010 – unpublished data) findings indicated increased SIgA levels after a multi-strain probiotic administration that was consistent with two other studies (8, 14). Immunoglobulin A was the main immunoglobulin involved in host defense and the IgA level appeared to be suppressed through physical training, or in situations of acute and intense stress, such as in chronic training of athletes. Probiotic supplementation in athletes showed no significant level changes due to chronic stress (15).

In this study, we obtained different results concerning a change in SIgA levels pre- and post-probiotic administration in the groups receiving a single-strain probiotic of either *Bifidobacterium* and *Lactobacillus*. In the *Bifidobacterium* group, there was a higher increase in SIgA levels than in the *Lactobacillus* group, although this was not statistically significant. In the *Lactobacillus* group, the SIgA levels were close to those of the placebo group, but this finding was also not statistically significant.

In one review, a single-strain probiotic was noted to be inferior to a multi-strain probiotic in terms of preventing infection and reducing pathogenic bacterial colonization (16). To maintain the gut barrier, a more complex interaction is needed between the ecologic balance of normal intestinal microflora, normal immune function, and intact epithelial cells (17). However, it is not clear how a multi-strain probiotic outperforms a single-strain probiotic.

The collective intestinal microbiome contains ten times more cells than the human body, and has been estimated to contain at least 100 times more genes than the human genome, and may induce numerous populations of immune cells (18, 19). A multi-strain probiotic may have a better opportunity to maintain or restore the intestinal microbiome in order to stimulate a superior immune response.

Our study findings show that multi-strain probiotic administration increased intestinal immunity compared to single-strain probiotic administration in burns patients at our hospital Burns Centre. Probiotic administration is considered safe for burns patients, and we recommend caution regarding adverse events for those undergoing long-term administration, particularly for susceptible individuals (7, 20). However, in this study, probiotic administration was quantified according to fecal SIgA levels only. These levels represented only intestinal immunity and not systemic immunity against intestinal pathogens. Moreover, we did not observe a risk of infection outcome in the study patients and this is a further limitation of the study.

## CONCLUSION

A single-strain probiotic supplementation of *Bifidobacterium* was found to be generally superior to *Lactobacillus* in increasing SIgA secretion-mediated intestinal immunity in the burns patients in this study; however, this finding was not statistically significant. Based on both studies undertaken at our institution, a multi-strain probiotic is recommended as a treatment modality for burns patients.
